# EBV Latent Membrane Protein 1 Activates Akt, NFκB, and Stat3 in B Cell Lymphomas

**DOI:** 10.1371/journal.ppat.0030166

**Published:** 2007-11-09

**Authors:** Kathy H. Y Shair, Katherine M Bendt, Rachel H Edwards, Elisabeth C Bedford, Judith N Nielsen, Nancy Raab-Traub

**Affiliations:** 1 Lineberger Comprehensive Cancer Center, University of North Carolina at Chapel Hill, Chapel Hill, North Carolina, United States of America; 2 Department of Pathology and Laboratory Medicine, University of North Carolina at Chapel Hill, Chapel Hill, North Carolina, United States of America; 3 Department of Microbiology-Immunology, University of North Carolina at Chapel Hill, Chapel Hill, North Carolina, United States of America; Washington University School of Medicine, United States of America

## Abstract

Latent membrane protein 1 (LMP1) is the major oncoprotein of Epstein-Barr virus (EBV). In transgenic mice, LMP1 promotes increased lymphoma development by 12 mo of age. This study reveals that lymphoma develops in B-1a lymphocytes, a population that is associated with transformation in older mice. The lymphoma cells have deregulated cell cycle markers, and inhibitors of Akt, NFκB, and Stat3 block the enhanced viability of LMP1 transgenic lymphocytes and lymphoma cells in vitro. Lymphoma cells are independent of IL4/Stat6 signaling for survival and proliferation, but have constitutively activated Stat3 signaling. These same targets are also deregulated in wild-type B-1a lymphomas that arise spontaneously through age predisposition. These results suggest that Akt, NFκB, and Stat3 pathways may serve as effective targets in the treatment of EBV-associated B cell lymphomas.

## Author Summary

Epstein-Barr virus (EBV) is linked to the development of multiple cancers, including post-transplant lymphoma, Hodgkin disease, and nasopharyngeal carcinoma. Latent membrane protein 1 (LMP1) is expressed in many EBV-associated cancers and is responsible for most of the altered cellular growth properties that are induced by EBV infection. This study reveals that LMP1 induces lymphomas in B-1a lymphocytes, a cell type that is susceptible to transformation in aged mice. The lymphomas require Akt, NFκB, and Stat3 signaling for enhanced growth and survival. The activation of the Stat3, Akt, and NFκB signaling pathways likely underlies the ability of LMP1 to promote malignant transformation.

## Introduction

Epstein-Barr virus (EBV) is a ubiquitous γ-herpesvirus that infects humans predominantly at an early age with greater than 90% of the adult population infected with EBV [1]. EBV is linked to the development of both B lymphocyte and epithelial cell malignancies, including Burkitt lymphoma, Hodgkin disease (HD), and nasopharyngeal carcinoma (NPC), and cancers linked to immunosuppression, including post-transplant lymphoma and AIDS-associated lymphomas [2,3]. In vitro infection of B lymphocytes with EBV induces permanent growth transformation, and this ability to affect cell growth regulation likely contributes to the development of cancer.

Many of the viral proteins expressed in transformed cells, including the EBV nuclear antigens and latent membrane proteins, have profound effects on cell growth regulation and are required for EBV latent infection and B cell transformation [1]. Latent membrane protein 1 (LMP1) is considered the major oncoprotein of EBV, as it transforms rodent fibroblasts to tumorigenicity in nude mice and is expressed in HD, NPC, and immunosuppression-associated tumors [4–8]. In B lymphocytes, LMP1 mimics CD40 signaling, and both LMP1 and CD40 are essential for EBV-mediated B cell transformation [9–11]. While CD40 interacts with CD40 ligand expressed on activated T cells to induce B cell activation and differentiation, LMP1 acts as a constitutive signal through ligand-independent oligomerization. LMP1 and CD40 interact with the same tumor necrosis factor receptor–associated factors (TRAFs) leading to activation of NFκB, c-Jun N terminal kinase (JNK), and p38 MAPK signaling pathways [12–16]. Activation of NFκB is required for EBV-induced B cell transformation and its inhibition rapidly results in cell death [17,18]. Recent studies indicate that LMP1 also activates phosphatidylinositol 3 kinase (PI3K)/Akt signaling and that this activation is required for LMP1-mediated transformation of rodent fibroblasts [5,19].

In vitro, primary B cells can be maintained by CD40 ligation in combination with IL4 treatment. In vivo, CD40 signaling is necessary for germinal center (GC) formation such that mice deficient for CD40 or CD40L are unable to form GCs in response to T cell–dependent antigens [20,21]. Both the membrane proximal and distal cytoplasmic regions of CD40 that bind TRAF6 and TRAFs2/3/5, respectively, are necessary for GC formation, but either region is sufficient to induce extrafollicular B cell differentiation and restore low affinity antibody production [22]. Functionally, LMP1 can rescue CD40-deficient mice and restore immunoglobulin (Ig) class switching, most likely because LMP1 recruits similar TRAF molecules, TRAFs 1/2/3/5 and TRAF6, through the C-terminal activation regions 1 and 2 domains, respectively. However, LMP1 is unable to restore affinity maturation and GC formation [23].

Several EBV transforming proteins have been studied in transgenic mouse models, however, only LMP1 induces tumor development when expressed under the control of the Ig heavy chain promoter and enhancer [24–26]. The LMP1 transgenic mice (IgLMP1) express LMP1 in B lymphocytes, and in mice older than 12 mo, lymphoma develops with increased incidence (40%–50%) compared to wild-type control mice (11%), suggesting that LMP1 contributes to tumor development [26]. The LMP1 lymphomas have rearranged Ig genes and have activated Akt, JNK, p38, and NFκB, with specific activation of the NFκB family member cRel [27].

In this study, the LMP1 transgenic lymphocytes and lymphomas were further characterized and their growth properties in vitro were determined. To obtain pure populations of malignant lymphocytes and to enable more detailed biochemical analyses, examples of primary lymphomas were inoculated and passaged in SCID mice. Interestingly, lymphoma development was restricted to B-1a lymphocytes, a self-replenishing population of cells that are prone to malignancy [28,29]. LMP1 transgenic lymphocytes had increased viability in vitro and viability was increased by the addition of IL4. In contrast, both LMP1-positive and -negative lymphoma cells were independent of IL4 co-stimulation for survival and proliferation in vitro with a complete absence of activated Stat6, the IL4 target. The lymphomas were also distinguished by constitutive activation of Stat3 and deregulation of the Rb cell cycle pathway. Inhibition of the PI3K/Akt, NFκB, and Stat3 signaling pathways blocked the enhanced growth of both LMP1 transgenic and malignant lymphocytes, suggesting that these pathways are required for their growth and survival. These appear to be the same targets that are deregulated in wild-type B-1a lymphomas that arise spontaneously through age predisposition. This study reveals that LMP1 promotes malignancy in cells with the inherent ability to proliferate and that the Akt, NFκB, and Stat3 signaling pathways are required for its growth stimulatory effects.

## Results

### High Levels of LMP1 Expression Correlates with the Development of Lymphoma

**Figure 1:**
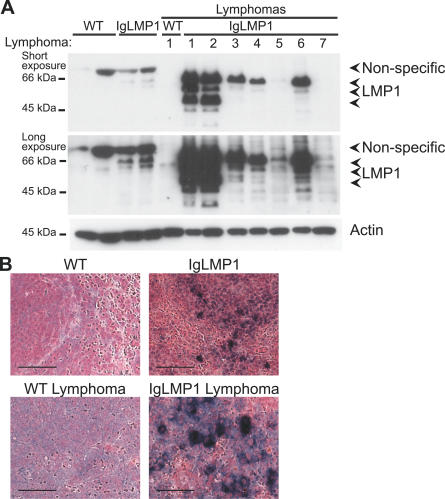
High LMP1 Expression Correlates with the Development of Lymphoma LMP1 expression is shown by (A) immunoblotting of purified B cells (CD19+) and (B) immunohistochemistry staining of spleen tissue from wild-type (WT) and LMP1 transgenic mice. (A) Lymphomas are identified with a number (1–7). Arrows indicate the LMP1-specific band and its degradation products as well as a non-specific band. Actin was used as a loading control. (B) White and red pulps are shown, but this architecture is lost upon development of lymphoma. Scale bar, 20 μm.

LMP1 expression in IgLMP1 mice was directed to B cells under the control of the Ig heavy chain promoter and enhancer. It has previously been shown that in these transgenic mice, LMP1 expression was restricted to B220+ B cells with lymphoma detected in greatly enlarged spleens [23,26]. To investigate whether LMP1 expression contributes to lymphoma development, B cells were purified from splenocytes by positive selection using anti-CD19 MACS magnetic beads, and equivalent amounts of B cells were analyzed by immunoblotting. LMP1 was detectable in LMP1 transgenic B cells, but upon development of lymphoma, LMP1 expression was stronger in 5/7 lymphomas analyzed with concomitant appearance of degradation products (Figure 1A). To determine whether the higher level of LMP1 detected was due to an expansion of malignant lymphocytes, expression of LMP1 in the spleen was further evaluated by immunohistochemical staining. Immunohistochemistry analysis of spleen sections detected LMP1 in the plasma membrane of cells in both the follicular white pulp and circulating lymphocytes in the red pulp (Figure 1B). LMP1 expression was heterogeneous with strong LMP1 staining interspersed amongst a background of cells staining weakly for LMP1. Upon development to lymphoma, LMP1 expression was more abundantly detected with multiple foci of intense LMP1 staining. This demonstrates that the increased LMP1 detected by immunoblotting upon malignant progression reflects an increase in LMP1 expression and an accumulation of cells expressing high levels of LMP1. This correlation between high LMP1 expression and the development of lymphoma suggests that progression to lymphoma results from increased levels of LMP1.

### LMP1 Promotes B-1a Lymphomas That Can Escape Allelic Exclusion

**Figure 2:**
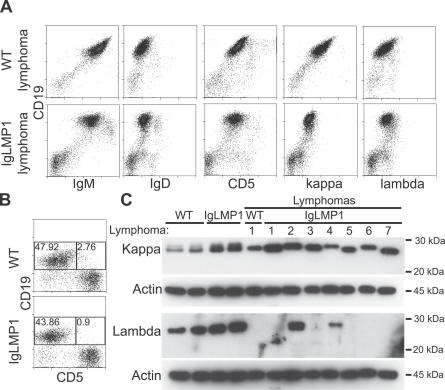
LMP1 Promotes B-1a Lymphomas That Can Escape Allelic Exclusion (A) Flow cytometry analysis of splenocytes from a WT or LMP1 transgenic lymphoma for the pan–B cell (CD19), B-1a cell (CD5), and Ig heavy chain (IgM and IgD) and light chain (κ and λ) markers. Shown are the results from WT lymphoma 1 and LMP1 transgenic lymphoma 4. This analysis was repeated on four other LMP1 transgenic lymphomas (1, 2, 3, and 6) showing a similar B-1a phenotype, of which lymphomas 2 and 4 were also doubly positive for κ and λ light chains. (B) Flow cytometry analysis of WT or LMP1 transgenic splenocytes for B-1a (CD19+CD5+) and B-1b or B2 subsets (CD19+CD5−). Percentages of B-1a and B-1b or B2 subsets are shown in each quadrant. This analysis was repeated on three other WT and two other LMP1 transgenic mice with similar results. (C) Immunoblot analysis for κ and λ light chains of B cells (CD19+) purified from WT and LMP1 transgenic mice. Actin was used as a loading control.

To determine if LMP1 signaling affects B cell differentiation and to immunophenotype the lymphomas that arise from LMP1 expression, surface Ig expression of heavy chains (IgM, IgG, IgD) and light chains (κ, λ) were analyzed by flow cytometry. Similar numbers of naïve (IgM+IgD+IgG−) splenic B cells with a strong bias towards κ light chain were detected from wild-type or LMP1 transgenic mice, indicating that LMP1 signaling does not affect B cell maturation (unpublished data). Flow cytometry analysis of the SCID-passaged wild-type and LMP1 transgenic lymphomas revealed an IgM^high^IgD^low^ phenotype (Figure 2A), indicative of marginal zone, B-1, or memory B cells. B-1 cells are further separated into CD5+ (B-1a) and CD5− (B-1b) subsets. To differentiate between these cell types, lymphoma cells were further analyzed for the B-1a marker CD5. All (5/5) of the tested LMP1 transgenic lymphomas displayed an IgM^high^IgD^low^CD5+ phenotype (Figure 2A), an expression pattern that distinguishes B-1a cells. Interestingly, a spontaneous wild-type lymphoma also developed in B-1a cells (Figure 2A). These cells are an interesting population that is self replenishing with an increased likelihood to become malignant in aged mice [30]. Analysis of LMP1 transgenic mice before the development of lymphoma showed similar numbers of splenic B-1a (CD19+CD5+) and B-1b or B-2 populations (CD19+CD5−), indicating that LMP1 does not affect B cell differentiation (Figure 2B).

Due to allelic exclusion, mature B cells that have been exposed to antigen will typically express only one heavy chain isotype (IgG, IgE, or IgA) and either a κ or λ light chain. Interestingly, 2/5 LMP1 transgenic lymphomas analyzed (lymphomas 2 and 4) were doubly positive for low levels of both κ and λ light chains (Figure 2A). Previous characterization of the LMP1 lymphomas had revealed that the lymphomas were clonal as determined by Ig heavy chain rearrangement [26], and analysis of κ chain rearrangement (Figure S1) of the samples analyzed in this study confirmed clonality. To further assess light chain expression, the passaged samples were tested by immunoblotting for κ and λ light chains (Figure 2C). Interestingly, very low levels of expression of both light chains were detected by flow cytometry. The low levels of expression may reflect a limitation of the total number of light chains that can be expressed on the surface of a B cell. In agreement with the flow cytometry analysis, LMP1 transgenic lymphomas 2 and 4 were also positive for κ and λ light chains by immunoblot analysis (Figure 2C), confirming that these lymphomas express both light chains. A previous study of mice that developed leukemia due to an expansion of self-reactive B-1a cells determined that the B-1a leukemias were also doubly positive for κ and λ light chains [31]. These findings indicate that expression of LMP1 in B-1a cells promotes the development of malignancy and can result in the aberrant escape from allelic exclusion.

### LMP1 Promotes B Cell Survival and Proliferation In Vitro

**Figure 3:**
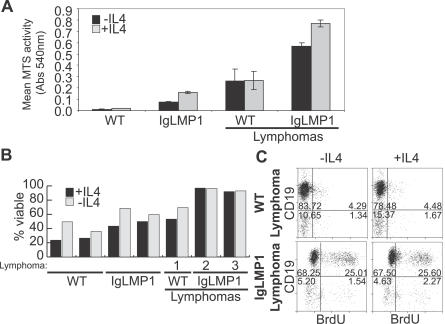
LMP1 Promotes B Cell Survival and Proliferation In Vitro (A) MTS assay of splenocytes from WT and LMP1 transgenic mice. Splenocytes were cultured in the presence (grey bars) or absence (black bars) of IL4 for 3 d. The results are the mean ± SEM of triplicate samples averaged from multiple mice where “n” the number of mice analyzed is as follows: *n* = 2 for WT lymphocytes and WT lymphomas, *n* = 11 for LMP1 transgenic lymphocytes, and *n* = 13 for LMP1 transgenic lymphomas. (B) EMA exclusion of CD19+ gated splenocytes from WT and LMP1 transgenic mice showing percentage of viable B cells cultured with (white bars) or without (black bars) IL4 for 2 d. (C) Flow cytometry analysis for incorporated BrdU in WT and LMP1 transgenic lymphoma cells cultured with or without IL4 for 2 d. Shown are the results from WT lymphoma 1 and LMP1 transgenic lymphoma 2. Percentages of cells in each quadrant are shown.

Primary B cell cultures can be maintained through CD40 ligation and supplementation with IL4 [32]. To investigate whether LMP1 affects primary B cell survival and proliferation, splenocytes were cultured in the presence or absence of IL4 and analyzed by MTS as a metabolic marker, by ethidium monoazide (EMA) exclusion for viability, and by 5-bromo-2′-deoxy-uridine (BrdU) incorporation for proliferation. In the MTS assay, as expected, splenocytes from wild-type mice did not survive even with the addition of IL4 due to a lack of CD40 ligation (Figure 3A). In contrast, LMP1 splenocytes had increased metabolism even in the absence of IL4, which was further enhanced upon addition of IL4. Wild-type and LMP1 transgenic lymphoma cells had high levels of MTS activity even in the absence of IL4 (Figure 3A). The LMP1 transgenic lymphoma cells had approximately 4-fold higher MTS activity than the normal transgenic lymphocytes and were at least 2-fold higher than the control lymphoma. As previously published, lymphoma usually develops in mice over 12 mo of age and all mice are sacrificed by 18–20 mo. The ages of the transgenic mice with or without lymphoma ranged between 6 and 20 mo old. There was no correlation between age and MTS activity.

EMA exclusion of CD19+ gated B cells prepared from two wild-type and two LMP1 transgenic mice indicated a 2-fold increase in viability in the LMP1 transgenic lymphocytes compared to wild-type lymphocytes. Two examples of LMP1 transgenic lymphoma cells had greatly increased viability that was not increased by IL4 treatment, indicating that the lymphoma cells are independent of IL4 co-stimulation (Figure 3B). Enhancement in MTS activity was observed in LMP1 transgenic lymphoma cells by the addition of IL4; however, EMA exclusion did not reveal a similar increase. This could reflect a difference for IL4 requirement in the metabolic activity versus the viability of LMP1 transgenic lymphoma cells. Although expression of LMP1 could enhance survival of non-malignant primary lymphocytes, BrdU incorporation revealed that LMP1 expression alone was not sufficient to induce proliferation in culture (unpublished data). Only lymphoma cells had detectable levels of BrdU incorporation detected by flow cytometry (Figure 3C). Interestingly, LMP1 lymphoma cells had significantly higher levels of proliferation in comparison to the spontaneous lymphoma that developed in an LMP1-negative littermate (25% versus 4%). This higher level of proliferation was observed in lymphomas that express both high (Table 1, LMP1-L2 and LMP1-L3) and low (Table 1, LMP1-L5) levels of LMP1, suggesting that even small amounts of LMP1 is sufficient to induce dramatic effects in proliferation. The level of proliferation was not enhanced upon IL4 addition, confirming the IL4 independence observed in the viability studies (Figure 3C; Table1).

**Table 1:**
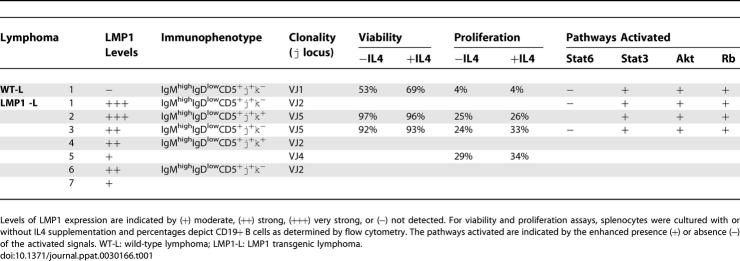
Summary of Analysis Performed on Wild-Type and LMP1 Transgenic Lymphomas

### Wild-Type and LMP1 Transgenic Lymphoma Cells Do Not Require IL4 and Stat6 Signaling

**Figure 4:**
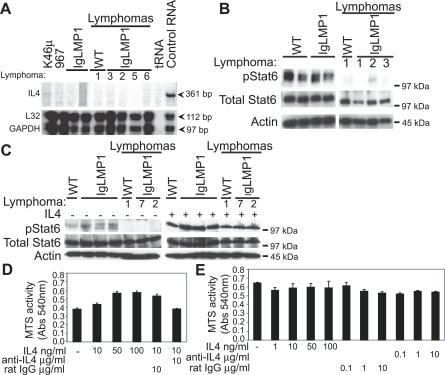
Wild-Type and LMP1 Transgenic Lymphoma Cells Survive Independently of IL4/Stat6 Signaling in Culture (A) Rnase protection assay for IL4 mRNA from purified B cells (CD19+) from WT and LMP1 transgenic splenocytes. The L32 and GAPDH housekeeping genes were used as a loading control. Arrow indicates the position of the protected probe. (B and C) Immunoblot analysis of WT and LMP1 transgenic mice for activated pStat6 in (B) purified B cells (CD19+) at the time of harvest or in (C) whole splenocytes cultured with or without IL4. (B) Actin was used as a loading control, and the white line indicates that intervening lanes have been spliced out. (D and E) MTS assay of (D) WT lymphocytes and (E) LMP1 transgenic lymphoma cells cultured with IL4, a neutralizing antibody to IL4, or a rat IgG isotype control at the indicated concentrations. Shown are the results from LMP1 transgenic lymphoma 3. The results are the mean ± SEM of triplicate samples from a single representative experiment that was repeated twice with similar results.

To investigate whether IL4 independence was due to endogenous IL4 expression, IL4 transcription was assessed by an Rnase protection assay (RPA). IL4 transcription was detectable with control RNA and faintly in the mouse lymphoma cell line K46μ (Figure 4A). However, IL4 transcription was not detectable in CD19+ MACS-purified B cells from wild-type lymphocytes (unpublished data), LMP1 transgenic lymphocytes, or lymphoma cells, although the GAPDH and L32 controls were effectively protected (Figure 4A). Activated Stat6 (pStat6), a target of the IL4 receptor pathway, was detected in the wild-type and LMP1 transgenic lymphocytes (Figure 4B). In contrast, pStat6 was barely detected in either the wild-type or LMP1 transgenic lymphoma cells. However, the pathway was not disabled, as treatment of the lymphoma cells with IL4 induced Stat6 phosphorylation (Figure 4C).

Although wild-type lymphocytes cannot be maintained in culture with IL4 supplementation alone (Figure 3A), slight enhancement in MTS activity could be detected if the cells were analyzed at an earlier time point, at 1 d (Figure 4D) versus 3 d (Figure 3A) post-harvest. The enhancement of MTS activity induced by IL4 in wild-type lymphocytes could be neutralized by the addition of IL4 antibody (Figure 4D). However, neutralizing antibodies to IL4 did not affect the MTS activity of LMP1 transgenic lymphoma cells (Figure 4E). In summary, the wild-type and LMP1 transgenic lymphoma cells grew independently of IL4 treatment and did not require Stat6 signaling.

### LMP1 Upregulates IL10 and Constitutively Activates Stat3

**Figure 5:**
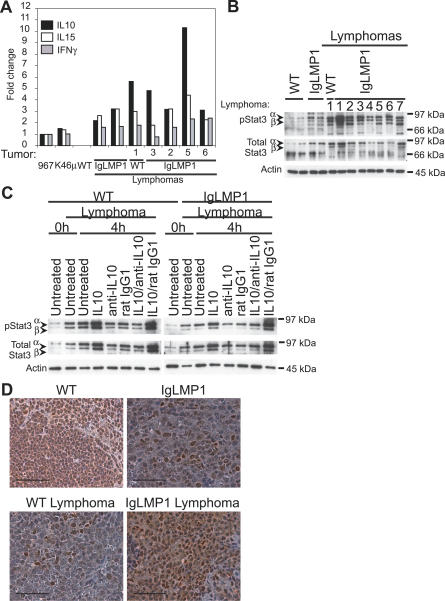
LMP1 Upregulates IL10 Expression and Constitutively Activates Stat3 (A) Relative expression of IL10, IL15, and IFNγ mRNA in WT and LMP1 transgenic B cells (CD19+), as detected with an Rnase protection assay. Mouse lymphoma cell lines 967 and K46μ were used as controls. Expression levels were quantified with a phosphorimager and values were normalized to the ribosomal housekeeping gene L32. The cytokine:L32 ratio was set to 1 in the mouse B cell lymphoma line 967. (B and C) Immunoblot analysis of activated pStat3 in purified B cells (CD19+) from WT and LMP1 transgenic mice (B) at the time of harvest, and (C) 4 h after culture with or without IL10, a neutralizing antibody to IL10, or a rat IgG1 isotype control. (C) Shown are the results for WT lymphoma 1 and LMP1 transgenic lymphoma 1. Arrows indicate the positions of the α and β isoforms of Stat3. Actin was used as a loading control. (D) Immunohistochemistry detection of activated nuclear pStat3 in the spleens of WT and LMP1 transgenic mice. Scale bar, 20 μm.

To identify cytokines that may contribute to the increased survival and growth of lymphomas, the expression levels of a panel of cytokines were screened on CD19+ MACS-purified B cells, using an RPA probe set for IL4, IL5, IL10, IL13, IL15, IL9, IL2, IL6, and IFNγ. Expression levels were quantified with a phosphorimager and normalized to the ribosomal housekeeping gene L32. None of the tested cytokines were detected in wild-type lymphocytes, therefore cytokine:L32 ratios were set to 1 in the mouse B cell lymphoma line 967. Transcription of IL10, IL15, and IFNγ were reproducibly detected in LMP1 transgenic lymphocytes and lymphoma cells and was higher than in the B cell lymphoma cell lines 967 and K46μ (Figure 5A). There was no significant difference in the expression of IL15 and IFNγ between LMP1 transgenic lymphocytes and lymphoma cells, suggesting that upregulation of IL15 and IFNγ is induced by LMP1 expression in healthy lymphocytes but is not a unique property of malignant lymphocytes. Strikingly, IL10, a B lymphocyte stimulatory cytokine, was increased 1.5- to 5-fold in the wild-type and LMP1 transgenic lymphoma cells compared to LMP1 transgenic lymphocytes (Figure 5A). Production of IL15 and IFNγ has been associated with induction of cytotoxic effector responses in cells latently infected with EBV [33,34]. However, transformation and growth properties induced by EBV are associated with the upregulation of IL10 [35–38]; hence, the effects of IL10 upregulation on the growth properties of the lymphoma cells were further examined. Immunoblot analysis indicated that LMP1 transgenic lymphocytes and wild-type and LMP1 transgenic lymphoma cells had corresponding increased levels of phosphorylated α and β isoforms of activated Stat3, a target of the IL10 receptor (Figure 5B). However, when comparing the same lymphomas, there was no correlation between the levels of IL10 induction and the levels of Stat3 activation. This suggests that the activation of Stat3 is not solely induced by IL10 or that Stat3 activation may be constitutive. Additionally, there was no correlation between the levels of LMP1 expression and the levels of IL10 induction (Figures 1A and 5A). This indicates that the induction of IL10 is a general property associated with enhanced survival and may only be indirectly affected by LMP1. Neutralizing antibodies to IL10 did not affect the survival of lymphoma cells as determined by the MTS assay (unpublished data), suggesting constitutive activation of Stat3. This was confirmed by immunoblot analysis such that in the presence of anti-IL10 neutralizing antibodies, pStat3 levels remained activated in lymphoma cells isolated from wild-type and LMP1 transgenic lymphomas (Figure 5C). Exogenous addition of IL10 enhanced pStat3 activation above constitutive levels, indicating that lymphoma cells are responsive to IL10 treatment (Figure 5C). This means that although the lymphoma cells have constitutive Stat3 activation, it may be further enhanced by IL10 induction. The neutralizing effect of the anti-IL10 antibody was confirmed by pre-incubation of IL10 with anti-IL10 antibody compared to a rat IgG1 isotype control (Figure 5C).

Nuclear translocation of pStat3 is a consequence of activation, and nuclear pStat3 was not detected by immunohistochemistry staining of spleen sections from control mice. However, nuclear pStat3 was detectable in LMP1 transgenic mice and wild-type lymphomas and was detected more homogeneously in LMP1 transgenic lymphomas (Figure 5D). The constitutive activation of pStat3 and abundant nuclear Stat3 suggests that Stat3 signaling contributes to LMP1-mediated lymphoma development.

### LMP1 Activates Akt Signaling and Deregulates the Rb Cell Cycle Pathway

**Figure 6:**
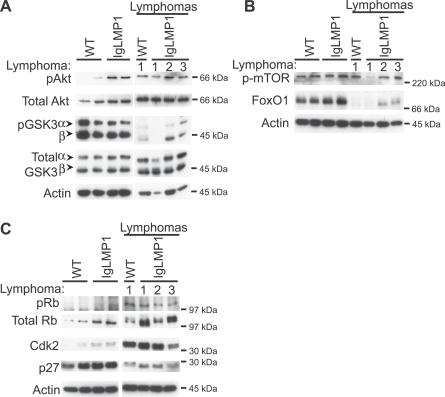
LMP1 Activates Akt Signaling and Deregulates the Rb Cell Cycle Pathway (A and B) Immunoblot analysis of purified B cells (CD19+) from the spleens of WT and LMP1 transgenic mice for Akt signaling, probing for (A) activated pAkt and downstream targets, including inactivated pGSK3α/β, and (B) activated p-mTOR, and total levels of FoxO1. Arrows indicate the positions of α and β isoforms of GSK3. The white line indicates that intervening lanes have been spliced out. (C) Immunoblot analysis for cell cycle proteins regulating the Rb pathway, probing for activated pRb, and total levels of Cdk2 and the Cdk inhibitor p27. Actin was used as a loading control.

LMP1 transformation of rodent fibroblasts requires activation of PI3K and Akt [5]. Additionally, activated pAkt is frequently detected in NPC and the neoplastic Reed-Sternberg cells of classical HD [39,40]. To determine if Akt signaling is activated in LMP1 transgenic mice, pAkt and several of its targets were assessed by immunoblotting of splenic CD19+ MACS-purified B cells. LMP1 transgenic B cells had increased levels of pAkt compared to wild-type lymphocytes; however, progression to lymphoma in both LMP1-positive and -negative lymphoma cells did not further increase pAkt levels. The Akt target glycogen synthase kinase 3 (GSK3) is inactivated by phosphorylation; however, increased phosphorylated GSK3 was not detected in the transgenic lymphocytes and was almost absent in the lymphoma samples (Figure 6A). This finding indicates that GSK3 is not a target of activated Akt in the LMP1 transgenic lymphocytes and lymphoma cells. Similarly, activation of Akt without phosphorylation of GSK3 has been previously shown in EBV-positive HD [40]. In contrast, the wild-type lymphocytes lacked activated Akt but did have detectable phosphorylated GSK3. This further suggests that additional pathways are involved in the regulation of GSK3.

To identify other potential Akt targets, immunoblot analysis for p-mTOR was performed. Activated p-mTOR was not increased in LMP1 transgenic lymphocytes or lymphoma cells, indicating that this pathway is not affected by LMP1-induced Akt activation and does not contribute to lymphoma development (Figure 6B). Akt is also known to phosphorylate and induce the degradation of the pro-apoptotic Forkhead family of transcription factors, leading to cell cycle progression and survival in some human tumors [41,42]. Immunoblot analysis of splenic B cells did not consistently detect p-FoxO1 levels, a signal that targets FoxO1 for degradation. Hence, degradation of FoxO1 was assessed by detection of total FoxO1 levels. Immunoblot analysis indicated that total FoxO1 levels were greatly decreased in wild-type and LMP1 transgenic lymphomas (Figure 6B), suggesting that inhibition of the Forkhead signaling pathway is an important target of Akt in lymphoma development. However, considering that Akt activation did not induce FoxO1 degradation in LMP1 transgenic B cells, Akt may not be the sole regulator of FoxO1, and it may be that progression to lymphoma requires modulation of multiple pathways.

The Forkhead family of transcription factors is known to induce the expression of the Cdk inhibitor p27 [43,44]. LMP1-transformed rodent fibroblasts have decreased expression of p27, upregulation of Cdk2, and subsequent phosphorylation and inactivation of the tumor suppressor gene Rb [45]. To investigate whether LMP1 affected cell cycle regulation through the Rb pathway in B cells, immunoblot analyses for pRb, Cdk2, and p27 were performed on splenic CD19+ MACS-purified B cells. LMP1 transgenic B cells had enhanced levels of pRb with concomitant stabilization of total Rb levels and Cdk2 compared to wild-type B lymphocytes (Figure 6C). Progression to lymphoma in both wild-type and LMP1 transgenic lymphoma cells led to increased levels of Rb, correspondingly high levels of Cdk2, and decreased levels of p27 (Figure 6C). These data indicate that the Rb pathway is deregulated in LMP1 transgenic lymphocytes and that lymphoma cells are distinguished by loss of FoxO1 and decreased p27.

### LMP1 Promotes Tumor Growth and Survival through Activation of Akt, NFκB, and Stat3 Pathways

**Figure 7:**
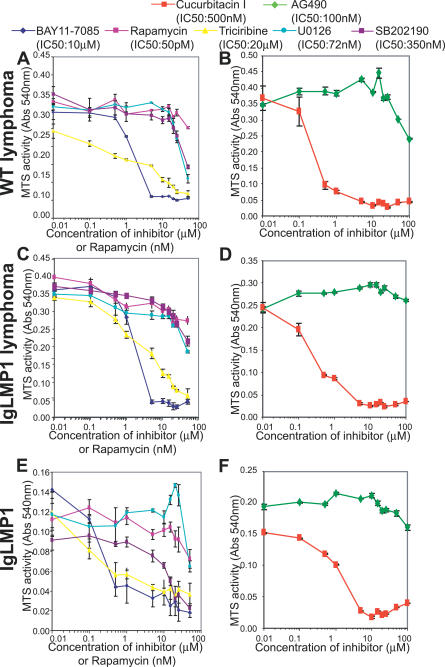
Akt, NFκB, and Stat3 Signaling Are Required for the Growth and Survival of Lymphoma Cells MTS assay of splenocytes from (A and B) WT or (C and D) LMP1 transgenic lymphomas and (E and F) LMP1 transgenic lymphoctyes. Splenocytes were cultured with or without inhibitors of NFκB (BAY11), mTOR (rapamycin), Akt (triciribine), MEK1/2 (U0126), p38 (SB202190), or Stat3 (cucurbitacin I and AG490) at the indicated concentrations. The results are the mean ± SEM of triplicate samples. Shown are the results for (A and B) WT lymphoma 1, (C and D) LMP1 transgenic lymphoma 2, and (E and F) one out of two LMP1 transgenic mice analyzed. This analysis was repeated with LMP1 transgenic lymphoma 4 yielding similar results.

**Figure 8:**
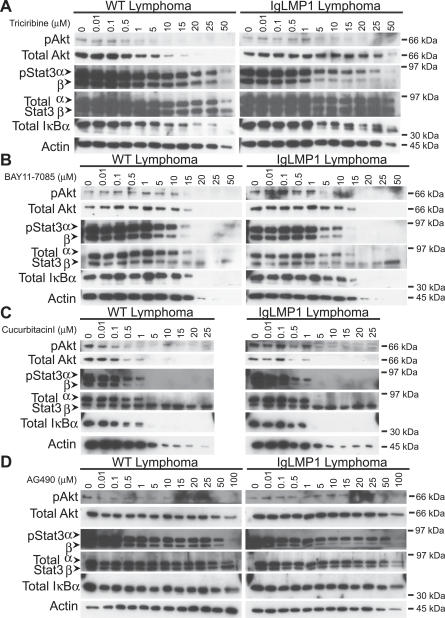
Analysis of Akt, NFκB, and Stat3 Pathways in Contribution to the Growth and Survival of Lymphoma Cells (A–D) Immunoblot analysis of wild-type and LMP1 transgenic lymphomas for Akt, NFκB, and Stat3 signaling after treatment with (A) an Akt inhibitor, triciribine, (B) an NFκB inhibitor, BAY11–7085, and the Stat3 inhibitors (C) cucurbitacin I and (D) AG490, at the indicated concentrations. Arrows indicate the positions of α and β isoforms of Stat3. Actin was used as a loading control.

To explore which pathways were required for the enhanced growth and survival of LMP1-induced lymphomas, splenocytes from wild-type and LMP1 transgenic mice were cultured in the presence of inhibitors for Akt, NFκB, Stat3, mTOR, or MAPK and assayed for growth and survival by the MTS assay. As previously shown, wild-type lymphocytes were not viable in culture and could not be tested with the inhibitors. However, the enhanced viability of LMP1 transgenic lymphocytes was effectively blocked by treatment with triciribine, BAY11–7085, cucurbitacin I, and slightly with SB203580, but not by treatment with rapamycin, U0126, or AG490 (Figure 7). Triciribine inhibits the activation of Akt and at 20 μM has been shown to induce growth arrest in cancer cells with aberrant Akt activity [46]. The effects of triciribine on cell growth of the transgenic lymphocytes and lymphomas were apparent as low as 1 μM, suggesting that activation of Akt is required for the survival and growth of LMP1 transgenic lymphocytes and lymphoma cells (Figure 7). The effects of the inhibitors were assessed by identifying phosphorylated Akt, Stat3, and total levels of IκBα (Figure 8). Treatment with triciribine effectively blocked phosphorylation of Akt, and phosphorylated Akt was no longer detected past 5 μM. Phosphorylated Stat3 and IκBα were still present at 25 μM. These findings suggest that triciribine specifically targets Akt and that Akt activation is required for the enhanced viability of the transgenic lymphocytes and lymphoma cells.

Inhibition of NFκB signaling rapidly induces cell death of EBV-transformed lymphocytes [17,18]. BAY11–7085, an inhibitor of NFκB signaling, also greatly decreased the viability of the LMP1 transgenic lymphocytes and lymphoma cells at doses as low as 1 μM, and at 5 μM the cells were completely nonviable (Figure 7). This is well within the reported IC50 of 10 μM. Phosphorylated Akt, Stat3, and total IκBα were still present up to treatment with 15 μM and then were no longer detected (Figure 8). This finding suggests that inhibition of NFκB can induce cell death in LMP1 transgenic lymphocytes and lymphoma cells without significant effects on activation of Akt or Stat3.

Cucurbitacin I inhibits activation of Stat3 by suppressing the activation of its kinase JAK2. It has been shown to selectively inhibit the growth of tumors with constitutively activated Stat3 [47]. Similarly, LMP1 transgenic lymphocytes and lymphoma cells were susceptible to cucurbitacin I treatment starting at 0.1 μM, a dose that corresponds closely to the reported IC50 of 500 nM (Figure 7) [47]. Phosphorylated Akt, Stat3, and total IκBα were not detectable past 1 μM and at higher doses all protein levels were greatly decreased, indicative of the total loss of viability (Figure 8). A second reported inhibitor of Stat3, AG490, had no effect on growth (Figure 7), but activation of Akt, Stat3, or levels of IκBα were also not affected (Figure 8). These findings suggest that inhibition of Stat3 can induce cell death in LMP1 transgenic lymphocytes and lymphoma cells, but Stat3 inhibition also has considerable crossover effects on Akt and NFκB signaling.

LMP1 has also been shown to activate JNK and p38 MAPK pathways [13,48], and LMP1 transgenic lymphocytes were mildly susceptible to growth inhibition by SB202190, an inhibitor of p38 MAPK, but not U0126, an inhibitor of MEK1/2 activity. However, effects of SB202190 were only apparent at high doses (>10 μM), much higher than the reported IC50 of 0.35 μM, suggesting that p38 MAPK does not significantly contribute to the enhanced viability in LMP1 transgenic lymphocytes or lymphoma cells (Figure 7). Interestingly, both wild-type and LMP1 transgenic lymphomas were similarly susceptible to triciribine, BAY11–7085, and cucurbitacin I treatments, but not SB202190, AG490, or U0126 treatment, suggesting that activation of Akt, NFκB, and Stat3 but not MAPK pathways are characteristics associated with malignant transformation (Figure 7). rapamycin, an inhibitor of mTOR, did not affect the viability of the transgenic lymphocytes or lymphoma cells, confirming that mTOR is not targeted by Akt activation in LMP1 transgenic lymphocytes or malignant lymphoma cells (Figure 7).

## Discussion

This study defines the oncogenic properties of LMP1 in promoting B cell lymphomagenesis. LMP1 transgenic mice have a higher incidence of lymphoma [26] and the progression to lymphoma correlates with higher expression levels of LMP1 (Figure 1A and 1B), suggesting that LMP1 is directly involved in tumor development. Table 1 summarizes the biological and molecular properties that were identified in wild-type and LMP1 transgenic lymphomas. Although many of the molecular properties studied were similar between wild-type and LMP1 transgenic lymphomas, there were distinguishing biological properties, namely the ability of LMP1 transgenic lymphomas to induce higher levels of survival and proliferation. Interestingly, although LMP1 transgenic mice develop lymphomas in the same B-1a cell type as spontaneous wild-type lymphomas (Figure 2), some signaling effects induced by LMP1 may explain the enhanced promotion to lymphomagenesis. Since CD40-deficient mice have decreased numbers of IgM^high^IgD^low^ cells, a phenotype associated with B-1, marginal zone, and memory B cells, the mimicry of CD40 signaling by LMP1 could possibly contribute to the expansion of B-1 cells [20]. It is noteworthy that expression of LMP1 in transgenic mice has been shown to inhibit the formation of GCs [23,49], preventing typical B-2 cells from antigen-driven selection and expansion. The lack of GC reactions may contribute to the bias of LMP1 transgenic mice towards B-1 cell lymphomas. Interestingly, LMP2 signaling also favors development of B-1 cells, but this occurs in the absence of transformation. These results suggest that the mimicry of B cell receptor signaling by LMP2 promotes B-1 cell differentiation but not transformation [24,50,51]. This promotion of B-1 differentiation may account for the ability of LMP2 to exacerbate autoimmunity and bypass anergy induction [52,53]. In contrast, the preponderance of tumors of B-1a origin does not reflect effects of LMP1 signaling on B cell differentiation, as splenic B cells from healthy LMP1 transgenic mice contain similar numbers of B-1 and B-2 cells as wild-type mice. In support of this lack of effect, the differentiation of B-1 versus B-2 cells is thought to be independent of CD40 signaling [54].

B-1 cells constitute the predominant lymphocyte population in the peritoneal and pleuropericardial cavities, while B-2 cells are mainly found in the spleen, lymph node, and peripheral blood. B-1 cells produce the main source of IgM and IgA antibodies in serum, which are involved in T cell–independent responses to common microbial antigens. Importantly, B-1 cells have the unique capacity to self replenish and are also predisposed to transformation [28,29]. Clonal expansion of B-1 cells can be detected in aging mice above 18 mo of age, and B-1 cells are thought to be the murine progenitor of B cell chronic lymphocytic leukemia [30]. The data presented in this study indicate that although LMP1 is expressed in all B lymphocytes in the transgenic mice, malignancy develops in this specific subset of B cells. The elevated expression of LMP1 in B-1a cells and the activation of specific pathways apparently induce malignant growth. These same pathways can also become sporadically activated in aged mice and also result in lymphoma development. This is similar to EBV-associated cancers in vivo, where pathways that are activated by LMP1 are also activated in the less prevalent EBV-negative forms of the cancers [40,55–58]. Thus, the contribution of EBV and LMP1 to tumor development is apparently the continuous activation of pathways that can also be sporadically activated and contribute to tumor development.

The lymphomas were marked by the upregulation of IL10, constitutive activation of Stat3 signaling, and a requirement for activation of Akt, NFκB, and Stat3 pathways (Figures 5 and 7). Induction of IL10 is associated with the transformation of B-1 lymphomas in mice [59,60] and is frequently associated with EBV-positive B cell malignancies acting as a B cell growth factor [35–38]. In addition, LMP1 has been shown to stimulate IL10 expression in Burkitt lymphoma cell lines [61,62]. This suggests that although Stat3 is constitutively activated in the lymphoma cells, the induction of IL10 may further enhance Stat3 activation or may contribute to other IL10-responsive signaling pathways.

LMP1 activates both the canonical and non-canonical pathways of NFκB signaling [14,63–65], and inhibition of NFκB blocked the survival of LMP1 transgenic lymphocytes and LMP1-positive and -negative lymphoma cells. NFκB and PI3K signaling are crucial for CD40-induced proliferation, and mice deficient for cRel or the p85 regulatory subunit of PI3K are unresponsive to mitogenic stimuli, including CD40 ligation [66–68]. We have previously shown that cRel is specifically activated in both wild-type and LMP1 transgenic lymphomas, suggesting that activation of cRel is associated with B cell transformation [27]. Our observations suggest that similar to CD40-induced proliferation, LMP1 induces proliferation through PI3K-mediated activation of Akt and activation of NFκB components such as cRel. CD40 also induces downregulation of the cell cycle inhibitor p27 through a PI3K-dependent manner, and the LMP1 lymphoma cells also had decreased levels of p27 with phosphorylation of Rb and increased Cdk2 (Figure 6C) [66]. Although LMP1 has been shown to deregulate the Rb pathway in epithelial cells [69], to our knowledge this is the first demonstration of this property in B lymphocytes.

The requirement for Akt activation was confirmed by the striking inhibition of lymphoma viability by triciribine, an Akt inhibitor. However, the activated pAkt did not lead to phosphorylation and inactivation of the downstream target GSK3 (Figure 6A). This effect has also been described in EBV-positive HD biopsies [40]. In contrast, rapamycin, U0126, and SB202190 did not affect the survival of LMP1 transgenic lymphocytes or the wild-type and LMP1 transgenic lymphoma cells (Figure 7A, 7C, and 7E). This lack of effect by rapamycin confirmed the absence of activated p-mTOR levels (Figure 6B). These findings suggest that other Akt targets contribute to malignant progression. One key target is likely the inhibition of the Fox01 transcription factors. Repression of the pro-apoptotic transcription factor FoxO1 in a PI3K-dependent manner can inhibit expression of bcl6, a transcription factor necessary for GC formation [49,70]. It has been shown that overstimulation of CD40 signaling with agonistic antibodies inhibits GC formation [71]. Similarly, due to mimicry of CD40 signaling, transgenic LMP1 mice are also defective in GC formation [23,49]. The constitutive signaling by LMP1 likely blocks GC formation through downregulation of bcl6. Interestingly, clinical studies indicate that expression of LMP1 and bcl6 are mutually exclusive in non-HD and classical HD [72,73]. Thus, the LMP1 transgenic lymphomas mirror aspects of EBV-induced HD. Although the activation of Akt and the lack of Fox01 in the lymphoma cells suggest that LMP1 affects bcl6 and GC formation through this pathway, regulation of other Forkhead targets involved in cell cycle progression, such as p27 and CyclinD2, likely contribute to malignant transformation. Indeed, loss of FoxO1 expression in lymphoma cells correlated with a loss of p27 (Figure 6B and 6C). CyclinD2 has also been shown to be upregulated by LMP1 through release of FoxO1-mediated repression [70].

In summary, in this transgenic model of lymphomagenesis, LMP1 promotes malignancy in B-1a cells, a population that is predisposed to clonal expansion with age. The malignant lymphocytes were distinguished by constitutively active Stat3 signaling, decreased p27, and activated Akt and NFκB pathways, properties that are associated with promoting the growth and survival of B lymphocytes. Importantly, Akt, NFκB, and Stat3 pathways were critically required for the growth and survival of malignant lymphocytes as well as healthy LMP1 transgenic lymphocytes. The growth of EBV-transformed lymphocytes also requires activation of NFκB, and these studies provide insight into how LMP1 contributes to EBV-associated transformation. The transgenic lymphomas mirror multiple aspects of EBV-induced tumors and suggest that in vivo these properties of LMP1 are major factors in the development of cancer.

## Materials and Methods

### Transgenic mice.

Generation of LMP1 mice under the Ig heavy chain promoter and enhancer have been described previously and were maintained as heterozygotes on a Balbc background [26]. LMP1 mice were genotyped by Southern blot and PCR analysis of tail DNA as described previously [26]. Spleen and liver sections were fixed in 4% paraformaldehyde and embedded in paraffin, and 5-μm sections were stained with hematoxylin and eosin for histopathological analysis. Lymphomas were passaged by intraperitoneal injection of 1 × 10^8^ splenocytes into SCID mice and sacrificed upon development of an extended abdomen. Animals were housed in the Association for Assessment and Accreditation for Animal Care–approved animal facility at the University of North Carolina at Chapel Hill. All protocols were approved by the Institutional Animal Care and Use Committee.

### Isolation and growth of B cells.

Splenocytes were prepared by homogenizing spleen tissue with two frosted slides and debris was filtered through a 100-μm cell strainer. Erythrocytes were lysed using 0.8% ammonium chloride solution (StemCell Technologies) for 10 min on ice and washed twice with PBS. B cells were isolated using CD19-MACS beads according to the manufacturer's instructions (Miltenyi Biotec) and grown in Iscove's medium supplemented with heat-inactivated 10% fetal bovine serum and antibiotic/antimycotic (GIBCO). Splenocytes isolated from SCID-passaged lymphomas consisted of 80%–90% B cells as determined by flow cytometry, and were hence not further purified with CD19-MACS beads. Splenocytes were seeded at 1.25 × 10^6^ cells/ml and where applicable, recombinant mouse IL4 was added at 100 ng/ml, recombinant mouse IL10 at 10 ng/ml, and rat IgG1 anti-mouse IL10 and rat IgG1 isotype control at 10 μg/ml (R&D Systems). For BrdU incorporation assays, splenocytes were pulsed for 24 h with 10 μM BrdU 1 d post-harvest.

### MTS assay.

The 3-(4, 5-dimethylthiazol-2-yl)-5-(3-carboxymethoxyphenyl)-2-(4-sulfophenyl)-2H-tetrazolium inner salt (MTS) cell cytotoxicity/proliferation assays were performed using the CellTiter 96 aqueous one-solution cell proliferation assay (Promega), according to manufacturer's instructions. For IL4 studies, splenocytes were cultured for 3 d in the presence or absence of 100 ng/ml IL4. Cells were seeded on day 3 in triplicate in a 96-well plate at 2.5 × 10^6^ cells/ml at 100 μl per well. MTS reagent was added for 4 h and absorbance was read at 540 nm; values plotted were subtracted from blanks. For neutralization assays, splenocytes were seeded at 5 × 10^6^ cells/ml at 100 μl per well on day of harvest and IL4, rat IgG1 anti-mouse IL4, or rat IgG1 isotype control (R&D Systems) were added at the concentrations indicated in the figures. Cultures were pulsed for 4 h with MTS reagent 1 d post-seeding. For inhibitor studies, splenocytes were seeded at 1 × 10^7^ cells/ml at 100 μl per well on day of harvest, and inhibitors were added at the concentrations indicated in the figures. The inhibitors BAY11–7085, rapamycin, triciribine, U0126, SB202190, and cucurbitacin I were purchased from EMD Biosciences. For non-malignant splenocyte cultures, B cell activation was induced with 10 μg/ml of goat F(ab') anti-mouse IgM (Jackson ImmunoResearch). Cultures were pulsed for 4 h with MTS reagent 1 d post-seeding.

### Immunohistochemistry.

Paraffin-embedded spleen sections were deparaffinized in Histoclear (National Diagnostics) and rehydrated in graded ethanol. Sections were antigen retrieved by microwaving in citrate buffer (pH 6.0) for 15 min (LMP1 staining) and 10 min (pStat3 staining). For LMP1 staining, sections were blocked with 1% BSA and 0.1% cold fish skin gelatin followed with streptavidin/biotin block (Vector Labs). Rat IgG anti-LMP1 (clones 8G3 and 1G6, Ascenion) were used at 1:10 dilution from tissue culture supernatants, followed with 8 μg/ml biotinylated mouse F(ab') anti-rat IgG (H+L) pre-adsorbed to mouse serum (Jackson ImmunoResearch) and 2 μg/ml streptavidin-alkaline phosphatase conjugate (Jackson ImmunoResearch). Stains were developed with BCIP/NBT and counterstained in Nuclear Fast Red (Dako). Phospho-Stat3 was detected with 4 μg/ml of pStat3 antibody (Tyr705, Cell Signaling) and detected with anti-rabbit Poly-HRP IHC detection kit (Chemicon).

### Flow cytometry.

One million splenocytes were stained with the appropriate primary antibody unconjugated or conjugated to FITC, PE, or APC diluted in stain buffer (PBS with 3% FBS). For BrdU detection, the FITC-BrdU flow kit was used as instructed by the manufacturer (BD Bioscience). Briefly, cells were exposed to EMA (Molecular Probes) for exclusion of dead cells, stained for surface antigens, fixed in paraformaldehyde, and permeabilized with saponin. To expose BrdU epitopes, cells were treated with Dnase and stained with FITC-conjugated anti-BrdU antibody. Flow cytometry was performed on FACScalibur using the CellQuest program (Becton Dickinson). Further analysis was conducted on the Summit v4.2 program (Dako).

### Rnase protection assay.

Total RNA was isolated from CD19+ MACS-purified B cells using the Rneasy midi purification kit with Dnase treatment, according to the manufacturer's instructions (Qiagen). The mCK-1 probe template set (BD Biosciences) was labeled using the In Vitro Transcription Kit according to the manufacturer's instructions (BD Biosciences). Briefly, 50 ng of mCK-1 probe set was labeled with [α-^32^P]UTP using T7 RNA polymerase and purified using Sephadex G-50 columns (NucAway Spin column, Ambion). The labeled probe was quantitated using a scintillation counter, and 6 × 10^5^ cherenkov cpm was used to hybridize to 4 μg of total RNA using the RPA kit (BD Biosciences). Samples were denatured at 90 °C and hybridized at 56 °C overnight. Single-stranded RNA was digested with a mixture of Rnase A and T1, and precipitated using isopropanol. Protected probes were resolved on a denaturing 4.75% acrylamide gel, dried, and imaged using a phosphorImager (Molecular Dynamics). Densitometry was performed using the ImageQuant TL v2005 software (GE Healthcare).

### PCR analysis of κ chain rearrangement.

DNA was isolated from splenocytes using the Dneasy Tissue Kit (Qiagen), with Rnase treatment. PCR reactions contained 100 ng of genomic DNA, 0.2 μM each primer, 0.2 mM dNTPs, and 2.5 U Taq DNA polymerase (NEB) performed in 1X ThermoPol buffer (NEB). Primers used were Vκcon and Jκ5–1° and have been described previously [74]. PCR conditions were 94 °C for 2 min, 40 cycles of 94 °C for 30 s, 63 °C for 90 s, and 72 °C for 1 min, followed by 1 cycle of 72 °C for 5 min.

### Immunoblot analysis.

Whole cell lysates were prepared in radioimmunoprecipitation assay (RIPA) buffer (20 mM Tris-HCl [pH 7.5], 150 mM NaCl, 1 mM EDTA, 1% NP-40, 0.1% SDS, 0.1% sodium deoxycholate) supplemented with 2 mM phenylmethylsulfonyl fluoride, 1 mM Na_3_VO_4_, and 1:100 protease/phosphatase inhibitor cocktails (Sigma). Crude lysates were centrifuged at 13,000 rpm for 10 min at 4 °C and the supernatants were collected for further analysis. Protein concentrations were determined with the Bio-Rad D_C_ protein assay system. Lysates were boiled in the presence of 2.5% β-mecaptoethanol, separated by denaturing SDS-PAGE, and transferred to 0.45-μm Optitran membranes (Schleicher & Schuell) in a Bio-Rad transfer unit. Membranes were immunoblotted with the appropriate primary antibody followed by horseradish peroxidase–tagged secondary antibodies (Amersham Biosciences and Dako) and detected with the SuperSignal West Pico System (Pierce).

### Antibodies.

FITC-conjugated goat anti-mouse IgM, rat IgG2aκ anti-mouse IgD (clone 11–26), rat IgG1κ anti-mouse kappa (clone 187.1), rat IgG2bκ anti-mouse lambda (clone JC5–1); PE-conjugated goat anti-mouse IgG; un-conjugated goat anti-mouse kappa and anti-mouse lambda were purchased from Southern Biotech. APC-conjugated rat IgG2aκ anti-mouse CD19 (clone 6D5), PE-conjugated mouse IgG2aκ anti-LMP1 (clone S12), un-conjugated mouse IgG2a anti-Rb (clone 2), and anti-Cdk2 (clone 55) were purchased from BD Bioscience. PE-conjugated rat IgG2aκ anti-mouse CD5 (clone 53–7.3) was purchased from eBioscience. Rat anti-LMP1 (clones 8G3, 1G6, 7E10, and 7G8) was purchased from Ascenion. Rabbit anti-pAkt (Ser473), anti-pGSK3α/β (Ser21/9), anti-pStat3 (Tyr705), anti-pStat6 (Tyr641), anti-pmTOR (Ser2448), anti-Stat6, anti-Akt, anti-FoxO1, and anti-p27 were purchased from Cell Signaling. Rabbit anti-Stat3 (H-190), anti-IκBα (C-21), and goat anti-β actin (I-19) were purchased from Santa Cruz Biotechnology. Mouse IgG1κ anti-GSK3 was purchased from Upstate Biotechnology. Rabbit anti-pRb (Thr373) was purchased from EMD Biosciences.

## Supporting Information

Figure S1 Lymphomas Are Clonal by κ Light Chain Rearrangement PCR analysis of total spleen genomic DNA for the mouse κ locus. Primers were designed to amplify all V-J rearrangements, including VJ1, VJ2, VJ4, and VJ5. Specific PCR products for the four rearrangements corresponding to the calculated sizes are indicated with arrows. Bands of other sizes are due to non-specific products. If a tumor was present in the background of normal spleen cells, a single band appears brighter than all the other specific bands. Wild-type and LMP1 transgenic lymphocytes are positive for all four V-J rearrangements and serve as positive controls. Clonality is determined by the appearance of a predominant band at the correct size for a single V-J rearrangement.
(739 KB PDF)

### Accession Numbers

The GenBank (http://www.ncbi.nlm.nih.gov/Genbank/) accession number for the EBV genome sequence is AJ507799. The gene identifier for LMP1 is BNLF1.

## Abbreviations

BrdU: 5-bromo-2′-deoxy-uridine
EBV: Epstein-Barr virus
EMA: ethidium monoazide
GC: germinal center
GSK3: glycogen synthase kinase 3
HD: Hodgkin disease
Ig: immunoglobulin
JNK: c-Jun N-terminal kinase
LMP1: latent membrane protein 1
NPC: nasopharyngeal carcinoma
PI3K: phosphatidylinositol 3 kinase
RPA: Rnase protection assay
TRAF: tumor necrosis factor receptor–associated factor
