# Supplementary material for: EBV Latent Membrane Protein 1 Activates Akt, NFκB, and Stat3 in B Cell Lymphomas
Source: PLoS Pathog. 2007 Nov 9;3(11):e166. doi: 10.1371/journal.ppat.0030166 (PMC2065877; doi:10.1371/journal.ppat.0030166)
Supplement: Supplementary file 1 [file ppat.0030166.sg001.pdf]

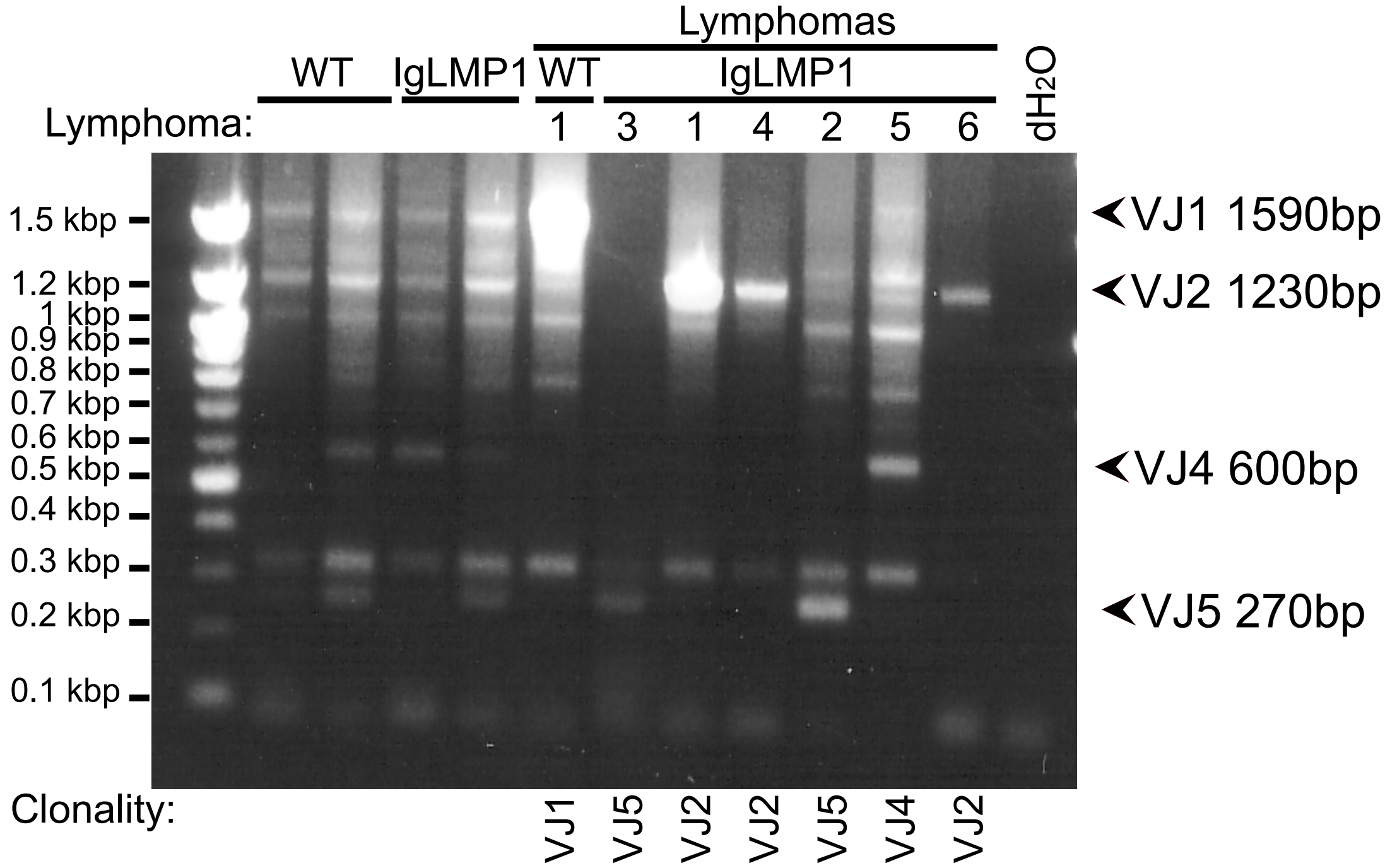
Lymphomas
WT
IgLMP1
WT
IgLMP1
Lymphoma:
1
3
1
4
2
5
6
dH2O
1.5 kbp
1.2 kbp
1 kbp
0.9 kbp
0.8 kbp
0.7 kbp
0.6 kbp
0.5 kbp
0.4 kbp
0.3 kbp
0.2 kbp
0.1 kbp
VJ1 1590bp
VJ2 1230bp
VJ4 600bp
VJ5 270bp
Clonality:
VJ1
VJ5
VJ2
VJ2
VJ5
VJ4
VJ2
